# The Evolution of Prostate Biopsy: A Retrospective Comparison of Transperineal and Transrectal Approaches Under Local Anaesthesia

**DOI:** 10.7759/cureus.103547

**Published:** 2026-02-13

**Authors:** Paul Lim, Amandeep Dosanjh, Meyada Ali, Mahmoud Mohammed, Ahmed Kodera, William Gallagher, Paul Rajjayabun

**Affiliations:** 1 Urology, Worcestershire Acute Hospitals NHS Trust, Redditch, GBR; 2 Surgical Oncology, South Egypt Cancer Institute, Assiut, EGY

**Keywords:** cancer detection rate, complications, prostate biopsy, prostate cancer, tolerability, transperineal, transrectal

## Abstract

Background and objective

First-line tissue sampling for prostate cancer can be performed using either transrectal ultrasound biopsy (TRUS) or local anaesthetic transperineal biopsy (LATP). The aim of this study is to compare these two techniques, examining outcomes of procedure tolerability, complications and cancer detection rate.

Methods

A retrospective, single-centre cohort study of patients undergoing prostate biopsy was performed. A telephone questionnaire was completed 72 hours post-biopsy, focusing on pain scores and complications. Cancer detection rates were also recorded.

Results

This study included 110 patients (55 LATP, 55 TRUS). Clinical data examining age, digital rectal examination findings, prostate-specific antigen (PSA), PSA density, prostate volume and prostate imaging reporting and data system (PI-RADS) score were gathered. Tolerability in both LATP and TRUS was similar. The median pain score on ultrasound probe use and biopsy gun deployment was marginally higher in the TRUS group. Difficulty passing urine occurred in 9% of LATP and 16% of TRUS (p=0.3916). Haematuria was seen in 58% of LATP and 73% of TRUS (p=0.1600). No patient required hospital admission. Cancer detection rates for LATP versus TRUS were 58.2% and 47.3%, respectively, (p = 0.3397) with higher LATP detection also seen in isolated PI-RADS 4/5 cases (p=0.2270). No statistically significant difference was found in detection rates or complications.

Conclusion

LATP is safe when compared to TRUS in all aspects of procedure tolerability, complications, and cancer detection rate.

## Introduction

Transrectal ultrasound biopsy (TRUS) has traditionally been utilised as the first-line modality for histologically diagnosing prostate malignancy, due to its tolerance by patients and the ability to perform it quickly under local anaesthesia (LA) in an outpatient setting.

TRUS has several recognised drawbacks, including poor targeting and suboptimal sampling of anterior lesions. Pre-biopsy multi-parametric magnetic resonance imaging (mpMRI) has now become the gold standard for prostate cancer care in the UK and worldwide [1,2]. This advancement has reshaped the diagnostic pathway, enabling better identification of patients at higher risk of clinically significant prostate cancer and allowing precise localisation of suspected lesions within the prostate [3]. It is understood that anterior lesions encompass approximately 20% of prostate cancers [4]. It is well established in the literature that transperineal (TP) biopsy is superior for sampling anterior lesions, with general cancer detection rates at least comparable to TRUS and some studies reporting superior outcomes [1,2,4-8].

Severe urosepsis is of key concern for morbidity and mortality, occurring in 2-3% of patients [9,10]. Although antibiotic prophylaxis is routinely used as a preventive measure, there are growing concerns about the rise of multidrug-resistant bacteria, which have heightened apprehension regarding the safety of TRUS biopsy [11,12]. The analysis of national Hospital Episode Statistics (HES) data by Tamhankar et al. [13] found that the rate of sepsis in TRUS biopsies has more than doubled over the decade (1.12% vs 0.53%). In addition to the associated morbidity and mortality, the economic burden to the health service is significant. It is estimated that readmissions secondary to TRUS-related urosepsis had an annual cost of £ 7.7-11.1 million in England and Wales [14]. TP biopsy as an alternative route of sampling avoids breach of the rectal mucosa, with current evidence suggesting a near-negligible readmission rate due to infection [15-17].

TRUS is increasingly being replaced by TP biopsy, with over a third of departments in the UK having adopted this method in 2019 [13]. TP biopsy was previously reserved only for specific cases and required general anaesthesia (GA). However, recent advances have made it possible to perform these biopsies under LA. The TRexit initiative [10], launched in 2020, has now evolved into a global project with the goal of establishing local anaesthetic transperineal biopsy (LATP) as the standard of care in prostate cancer investigation. The latest European Association of Urology (EAU) guidelines now recommend the use of TP biopsies due to lower risk of infections [1]. This is echoed by the National Institute of Clinical Excellence (NICE) recommendations, which also refer to the improved cancer detection rates in TP biopsy [2].

The aim of this study is to retrospectively examine tolerability, complications, and cancer detection rates of LATP as compared to TRUS biopsy.

## Materials and methods

Study design

This is a single-site, retrospective cohort study conducted between July 2022 and June 2023. A selection of patients undergoing prostate biopsy was identified, with half undergoing LATP biopsy and the other half TRUS biopsy. Allocation to each technique was determined by clinical availability during the department’s transition period to the TP approach, rather than by patient or surgeon preference. Both LATP and TRUS biopsies were performed by urologists who were independently competent in performing each procedure. Operator experience was standardised, as all procedures were conducted by clinicians trained and accredited in both techniques.

Patients were referred with an elevated prostate-specific antigen (PSA) level or abnormal digital rectal examination (DRE). Those deemed suitable for potential radical treatment underwent pre-biopsy mpMRI. Patients with Prostate Imaging Reporting and Data System (PI-RADS) ≥3 lesions were counselled and proceeded to biopsy if they consented, while those with non-suspicious MRI findings were discharged or followed up. PI-RADS classification was performed according to published international guidelines, which are freely available for clinical and academic use [18,19]. The study cohort therefore represents consecutive patients who met clinical and imaging criteria for biopsy during the study period.

Pain was assessed using a numerical rating scale (NRS), an 11-point ordinal scale ranging from 1 (no pain) to 10 (worst imaginable pain), which is a non-proprietary and freely available pain assessment tool widely used in clinical research [20]. Pain scores were recorded at predefined stages of the procedure: pre-operative prostate pain, local anaesthetic infiltration, ultrasound probe use, biopsy gun deployment, and prior to discharge. Patients were contacted 72 hours post-biopsy by telephone to determine their final pain score and any reported complications (such as difficulty passing urine, haematuria, infection & need for medical attention). If applicable, patients were also asked to compare their experience with any previous prostate biopsy.

Cancer detection rates were evaluated for the entire cohort and further analysed according to PI-RADS classification. A planned sub-analysis compared detection outcomes in patients with PI-RADS 3 lesions with those who had PI-RADS 4 or 5 lesions on pre-biopsy mpMRI. Any biopsy demonstrating prostate cancer, including International Society of Urological Pathology (ISUP) Grade Group 1 (Gleason score 3+3), was considered a positive result, as the aim was to assess overall cancer detection rather than restrict the analysis to clinically significant disease. Histopathological grading was performed using the International Society of Urological Pathology (ISUP) Grade Group system, an internationally accepted, consensus-based classification for prostate cancer that is widely implemented in clinical practice and research without licensing restrictions, and applied according to established published criteria [21]. The number of patients undergoing targeted biopsy was recorded, and within this group, the proportion of targeted cores yielding cancer was analysed.

Clinical and demographic data were obtained through the electronic portal system. This included age, DRE findings, PSA levels, prostate volume, PSA density, and PI-RADS score. Information on histopathological outcomes and the presence of any targeted lesions was also collected.

No formal sample size calculation was performed, as the study population was defined by the number of patients undergoing LATP and TRUS biopsies during the departmental transition period. The sample therefore reflects consecutive eligible patients treated over the study window. While the study was not powered to detect small between-group differences, it provides retrospective comparative data across two equally sized cohorts.

Descriptive statistics were used to summarise clinical and demographic data. Fisher’s exact test was used for categorical variables. Continuous variables were compared using the independent t-test or the Mann-Whitney U test for non-normally distributed data. All statistical tests were two-sided, with statistical significance defined as p < 0.05. All statistical methods used are non-proprietary and freely available.

This study was registered and authorised with the local audit department. Consent was obtained from patients to be included in the study.

Biopsy procedure

All biopsies were undertaken in a single outpatient setting. Additionally, all patients had urinalysis negative for infection on the day of biopsy.

TRUS Cohort

All patients were given a stat dose of intravenous (IV) gentamycin 160mg, oral ciprofloxacin 500mg and oral metronidazole 500mg (per rectum if the patient had a long-term catheter) as per the local departmental guideline. Patients were placed in the left lateral position. The ultrasound probe (BK Medical E14C4t Prostate Triplane Transducer) was inserted into the rectum to visualise the prostate. Peri-prostatic nerve block using 10ml of 1% lidocaine was administered bilaterally under ultrasound guidance, and 5 mins wait period was utilised for the anaesthetic to take effect. The ultrasound probe was then re-inserted into the rectum. The core biopsy device (Bard Max-Core™ Disposable Core Biopsy Instrument) was then introduced via the probe and directed into the prostate tissue. Generally, 12 TRUS guided systematic biopsy samples in total were obtained from the right and left sides, each requiring separate puncture through the rectal mucosa. When targeted biopsy was attempted, four cores were taken. Patients were discharged with further three-day course of oral ciprofloxacin.

LATP Cohort

No routine antibiotics were administered to patients undergoing LATP. Pre-procedural prophylactic antibiotics (intramuscular gentamicin 80 mg only) were only given to patients who were already catheterised or had a high post-void residual volume (>150mls) as per the departmental guideline. Patients were placed in the low lithotomy position and Elastoplast was used to elevate the scrotum away from the perineum. 10 ml of 1% lidocaine with adrenaline was administered to the skin around the needle entry sites. A biplanar transrectal ultrasound probe device (BK Medical E14CL4b Endocavity Biplane Transducer) mounted with PrecisionPoint® Transperineal Access System (BXT-Accelyon, Slough, UK) was inserted into the rectum to visualise the prostate. A peri-prostatic nerve block was performed bilaterally under ultrasound guidance using 20 ml of 1% plain lidocaine. After reinsertion of the probe, an access needle was introduced through the perineal skin. The core biopsy device was then introduced through this, and all cores were obtained through either one or two access sites on each side, depending on the size or position of the prostate. A total of 18 systematic cores were taken. This consisted of three cores from each of the six regions, with the regions divided into right and left sides and further subdivided into anterior, mid, and posterior zones. In cases of a targeted biopsy, four target cores were obtained.

## Results

Demographics

This study included a total of 55 patients who underwent TRUS and 55 patients who underwent LATP. Comparing the LATP group with TRUS, the median age was 71 years versus 69 years, respectively. There were 27/55 (49%) patients within the LATP group, who were found to have abnormal DRE compared to 24/55 (43.6%) patients in TRUS. For LATP and TRUS, respectively, the mean PSA was 12.8 ug/L versus 14.1 ug/L, the mean prostate volume was 59.0cc versus 58.7cc and the mean PSA density was 0.23 ug/ml versus 0.3 ug/ml. The median PI-RADS score was 4 in both groups. These demographic values are summarised in Table 1.

**Table 1:** Patient Demographics TRUS: Transrectal Ultrasound; LATP: Local Anaesthetic Transperineal Biopsy; DRE: Digital Rectal Examination; PSA: Prostate-Specific Antigen; PI-RADS: Prostate Imaging Reporting and Data System v2.1 [18,19]; IQR: Interquartile Range Continuous variables were compared using the independent t-test or Mann–Whitney U test where appropriate. Categorical variables were analysed using Fisher’s exact test.

Variable	LATP (IQR) (n=55)	TRUS (IQR) (n=55)	Test statistics	p-value
Median age	71 years (79-67)	69 years (78-64)	U=1292.5	0.2225
Abnormal DRE (%)	49.09 (n=27)	43.63 (n=24)	Fisher’s exact test	0.7024
Mean PSA (ug/L)	12.8 (11.5-4.6)	14.1 (11.4-4.6)	t=−0.31	0.7573
Mean prostate volume (cc)	59 (65.5-34)	58.7 (73.7-35)	t=0.04	0.9704
Mean PSA density (normal <0.15 ng/ml^2^)	0.23 (0.25-0.08)	0.3 (0.3-0.08)	t=−0.68	0.4983
Median PI-RADS [18,19]	4 (5-3)	4 (5-3)	U=1382	0.8887

Pain score

There was no statistically significant difference in the median numerical pain score between the two groups with regard to the baseline pre-operative pain (p=1), LA infiltration (p=0.3789) and immediate post-operative pain in recovery (p=0.9840). Patients undergoing TRUS reported higher pain scores for ultrasound probe insertion (p=0.0001) and biopsy gun deployment (p=0.0026). No patient in either cohort required the procedure to be abandoned. The median NRS values for peri-procedural and post-procedural pain are presented in Table 2.

**Table 2:** Median Numerical Rating Scale for Pain Peri & Post Procedure TRUS: Transrectal Ultrasound; LATP: Local Anaesthetic Transperineal Biopsy; IQR: Interquartile Range Pain scores were compared using the Mann–Whitney U test. Numerical rating scale [20]

	LATP (IQR) (n=55)	TRUS (IQR) (n=55)	Test statistics	p-value
Pre-operative prostate pain	1 (1-1)	1 (1-1)	U=1511.5	1
Local anaesthesia infiltration	3 (4-2)	3 (4-2)	U=1364	0.3789
Ultrasound probe use	2 (3-1)	4 (5-2)	U=865	0.0001
Biopsy gun deployment	1 (2-1)	2 (3-1)	U=987.5	0.0026
Prior to discharge following procedure	1 (1-1)	1 (1-1)	U=1508	0.9840
Current state (post 72 hours)	1 (1-1)	1 (1-1)	U=1375	0.4122

Complications and tolerability

In this study, 1/55 (1.8%) patient from the TRUS group developed a urinary tract infection (UTI), which was treated in the community with oral antibiotics following the procedure (Clavien-Dindo grade II). No cases of urosepsis or emergency hospital admissions were reported.

Reports of difficulty in voiding post procedure were more common in the TRUS group than in the LATP group: 16% (n=9) versus compared to 9% (n=5), respectively; this difference was not found to be statistically significant (p=0.3916). All cases resolved without catheterisation and were therefore classified as Clavien-Dindo grade I.

Haematuria was more common in the TRUS group (73% (n=40)) compared to the LATP group (58% (n=32)) (p=0.1600). Among those who reported haematuria, moderate to severe bleeding was observed in 6/32 (18.7%) of LATP patients compared to 12/40 (30.0%) of TRUS patients, although the difference was not statistically significant (p=0.4119). All were managed conservatively without transfusion or procedural intervention (Clavien-Dindo grade I). No grade III-V complications occurred in any of the patients included in this study.

All patients who underwent the LATP biopsy indicated they would be willing to repeat the procedure if necessary. There were 3/55 (5.5%) patients in the TRUS group that expressed a preference for alternative biopsy method, if they were to require another procedure. Of the LATP patients, 8/55 (14.5%) had undergone a previous prostate biopsy (7 via TRUS and 1 via a GA template biopsy). Notably, 7/8 (87.5%) of these patients reported a superior experience with LATP compared to their prior biopsies. In the TRUS group, 6/55 (10.9%) patients had a history of prior biopsy (1 via template and 5 via TRUS). These patients reported a similar experience with their current TRUS biopsy as with previous procedures.

Cancer detection

LATP achieved an overall cancer detection rate of 32/55 (58.2%), compared to 26/55 (47.3%) for the TRUS cohort. This difference was not statistically significant (p = 0.3397).

In the LATP group, 61.8% (n=34) of patients underwent targeted biopsies, compared to 43.6% (n=24) in the TRUS group, (p = 0.0852). Among the targeted biopsies, 19/34 (55.9%) were positive for cancer in the LATP group, compared to 10/24 (41.7%) in the TRUS group (p = 0.4242). This is summarised in Table 3.

**Table 3:** Cancer Detection Rate (Total Study) TRUS: Transrectal Ultrasound; LATP: Local Anaesthetic Transperineal Biopsy Categorical outcomes were analysed using Fisher’s exact test.

	LATP (n=55)	TRUS (n=55)	p-value
Cancer detection (%)	58.2 (n=32)	47.3 (n=26)	0.3397
Targeted biopsy (%)	61.8 (n=34)	43.6 (n=24)	0.0852
Positive target (%)	55.9 (n=19 out of 34)	41.7 (n=10 out of 24)	0.4242

The cohort was subdivided to determine the cancer detection rate based on their PI-RADS score which is also outlined in Table 4 and Table 5.

**Table 4:** Cancer Detection Rate (PI-RADS 3) TRUS: Transrectal Ultrasound; LATP: Local Anaesthetic Transperineal Biopsy; PI-RADS: Prostate Imaging Reporting and Data System v2.1 [18,19] Categorical outcomes were analysed using Fisher’s exact test.

	LATP (n=21)	TRUS (n=18)	p-value
Cancer detection (%)	33.3 (n=7)	38.8 (n=7)	0.7496
Targeted biopsy (%)	47.6 (n=10)	33.3 (n=6)	0.5158
Positive target (%)	10 (n=1 out of 10)	16.7 (n=1 out of 6)	1.0000

**Table 5:** Cancer Detection Rate (PI-RADS 4 and 5) TRUS: Transrectal Ultrasound; LATP: Local Anaesthetic Transperineal Biopsy; PI-RADS: Prostate Imaging Reporting and Data System v2.1 [18,19] Categorical outcomes were analysed using Fisher’s exact test.

	LATP (n=27)	TRUS (n=28)	p-value
Cancer detection (%)	81.4 (n=22)	64.2 (n=18)	0.2270
Targeted biopsy (%)	88.9 (n=24)	60.7 (n=17)	0.0286
Positive target (%)	75 (n=18 out of 24)	52.9 (n=9 out of 17)	0.1887

Amongst those who had LATP, 21/55 (38.2%) had PI-RADS 3 lesions compared to 18/55 (32.7%) who underwent TRUS. The cancer detection rates within this group showed LATP detected cancer in 7/21 (33.3%), while TRUS detected cancer in 7/18 (38.8%) with no statistically significant difference (p=0.7496). Targeted biopsies were performed in 10/21 (47.6%) of LATP cases, compared to 6/18 (33.3%) in the TRUS group, a difference that was not statistically significant (p=0.5158). Among these targeted biopsies, the positive detection rate was 1/10 (10%) for LATP and 1/6 (16.7%) for TRUS, with no statistically significant difference (p = 1.0000).

PI-RADS 4 and 5 lesions were observed in 27/55 (49.1%) LATP patients and 28/55 (50.9%) of those who received TRUS. Cancer was detected in 22/27 (81.4%) of LATP cases compared to 18/28 (64.2%) in the TRUS group. (p=0.2270). A statistically significant difference was identified in targeted biopsies: 24/27 (88.9%) of LATP patients versus 17/28 (60.7%) in the TRUS group (p = 0.0286). Among those who received targeted biopsies, LATP achieved a positive target rate of 18/24 (75%), compared to 9/17 (52.9%) in the TRUS group, (p = 0.1887).

## Discussion

The patient-reported outcome measures in this study indicated that LATP was generally well tolerated and provided greater comfort than TRUS, particularly regarding ultrasound probe insertion and biopsy gun deployment. Berquin et al. [16] also reported higher median pain scores for TRUS during biopsy sampling. LA infiltration was the most painful step in LATP, in line with observations by others [22,23]. This study directly associated tolerability with patient-reported pain scores; however, making comparisons across other studies was challenging due to variations in measurement tools. Lopez et al. [24] examined factors influencing tolerability beyond pain, finding that 6.8% of patients considered the procedure “very embarrassing”.

All patients successfully underwent both the LATP and TRUS, reflecting the low procedure abandonment rate of 0.37% reported by Kanagarajah et al. [23]. Notably, patients who underwent LATP stated they would be willing to repeat the procedure if needed. This strong level of acceptance aligns with the outcome from other studies where up to 90% of patients expressed similar willingness [22,25]. Collectively, these findings highlight LATP's acceptable tolerability and support it as a viable alternative to TRUS.

There were no reported readmissions for infection; the single case of post-biopsy infection occurred in the TRUS group, which was managed in the community, as compared with no diagnosed infections in the LATP cohort. No inferences can be made on urosepsis rates from the results of this study; however, current published evidence suggests that TP biopsy has a lower sepsis rate than TRUS [26,27].

Acute urinary retention (AUR) is a recognised risk of prostate biopsy, with approximately 25% of patients experiencing lower urinary tract symptoms [28]. The NPCA reported higher AUR-related readmission rates in LATP (1.93%) versus TRUS (0.95%) [27], while other studies yielded mixed outcomes [28,29]. In this analysis, urinary symptoms were more frequent after TRUS than LATP (16% vs. 9%). Haematuria is another common side effect, though severe cases requiring intervention are rare (<1%) [28]. Findings in this study indicated that haematuria was more frequent and severe in TRUS, but the difference was not statistically significant. Other reported incidence varies widely (2-84%), reflecting differences in methodologies and patient factors [12,16,27,29].

LATP demonstrated cancer detection rates comparable to TRUS, with an overall rate of 55.9%. This aligns with findings from previous studies, which reported detection rates ranging from 55% to 76.3% [5,24,25]. A meta-analysis by Uleri et al. [30] further highlighted significantly higher detection rates for PI-RADS 4 lesions with TP biopsy, but no significant differences were observed for PI-RADS 3 and 5 lesions. Our findings align with this, showing no significant difference in PI-RADS 3 detection rates between LATP and TRUS. However, for combined PI-RADS 4 and 5 lesions, LATP demonstrated a trend toward higher detection rates compared to TRUS (81.4% vs. 64.2%), though this was not statistically significant. Notably, in targeted biopsies, LATP achieved a 75% detection rate, outperforming TRUS at 52.9%.

This study has several strengths. Patient-reported outcome measures were systematically recorded as part of routine clinical practice, allowing a structured assessment of tolerability. The use of consistent biopsy protocols within each technique helped minimise procedural variability, enabling meaningful comparison between LATP and TRUS. In addition, the inclusion of both safety outcomes and cancer detection rates provides a balanced evaluation of diagnostic performance alongside patient-centred and clinical endpoints, strengthening the overall interpretability of the findings.

However, this study has several limitations. The relatively small cohort size in a single-centre study may limit the statistical power to assess the significance of outcomes. While the high tolerability and low complication rates in both cohorts are encouraging, further research with larger, multicentre studies is needed to validate these findings. Additionally, cancer detection rates were not further stratified into subgroups such as biopsy-naïve patients, those with prior negative biopsies, or patients under active surveillance, as done in some studies. Finally, variations in technical skills among clinicians performing the biopsies may have influenced the results through procedural variability.

## Conclusions

This study demonstrates that LATP is safe across all evaluated domains, including tolerability, complication rates, and cancer detection outcomes. Although further series of robust randomised controlled trials are required to establish comparative superiority, the increasing interest in evaluating the advantages of LATP highlights its potential to emerge as a widely accepted alternative to traditional TRUS biopsy. Overall, these findings support LATP as a promising approach in the diagnosis of prostate cancer.

## Appendices

LATP questionnaire and LATP STROBE checklist

https://drive.google.com/drive/folders/1j7kwjT5euMj4fQzDMMSOFMDeh1qT4Ev6?usp=sharing
